# Intravenous plus intravitreal erythropoietin for management of methanol toxicity optic neuropathy: A case report and brief review

**DOI:** 10.1002/ccr3.7011

**Published:** 2023-03-02

**Authors:** Mehran Rashidi Alavijeh, Sadegh Mazaheri‐Tehrani, Amir Sepehr Saffari, Mohammadreza Fazel, Farhad Fazel

**Affiliations:** ^1^ Student Research Committee, School of Medicine Isfahan University of Medical Sciences Isfahan Iran; ^2^ Isfahan Eye Research Center, Department of Ophthalmology Isfahan University of Medical Sciences Isfahan Iran

**Keywords:** erythropoietin, methanol, optic neuropathy, toxicity

## Abstract

Methanol can inhibit cellular aerobic respiration pathway and causes cell hypoxia specially in optic neurons. Despite using many drugs, methanol‐induced optic neuropathy (MION) still has a poor prognosis. Here we present a case of MION which is managed by a combination of intravenous and intravitreal erythropoietin in addition to corticosteroids.

## Abstract 2

Despite usage of many drugs, methanol induced optic neuropathy (MION) is still poor prognosis. Combination of intravenous and intravitreal erythropoietin in addition to corticosteroid can be an opportunity to treat MION.

## 1 INTRODUCTION

Methanol (methyl alcohol) is a clear, colorless, and flammable liquid, which is used in paint removers, photocopying fluids, and industrial solvents. In the human body, methanol is metabolized to its toxic metabolite, formic acid. Formic acid has an inhibitory effect on cytochrome C oxidase, an enzyme in the aerobic respiration pathway, and causes cell hypoxia. Therefore, cells like neurons that acquire their energy mostly from aerobic reactions are more vulnerable to hypoxia.^1^, ^2^, ^3^ So, methanol can lead to optic neuropathy through this mechanism.

Many types of drugs have been used to treat methanol‐induced optic neuropathy (MION) such as fomepizole, ethanol, B‐group vitamins, and corticosteroids. But the final visual acuity of the patients is still in the range of counting fingers, or even worse.^4^, ^5^, ^6^

Erythropoietin (EPO) is a glycoprotein that appears to have a neuroprotective and neurotrophic effect by decreasing apoptosis and inflammation, reducing the number of reactive oxygen species (ROS), and increasing progenitor cell proliferation, but its effect on MION is still unclear. Recent investigations declared that intravenous and subcutaneous EPO could be helpful to treat MION but there is no evidence about the effect of intravitreal EPO on this situation.^7^ Here, we present a case of optic neuropathy due to methanol toxicity, treated by combination of intravenous and intravitreal EPO in addition to corticosteroid, which showed a remarkable improvement in the visual outcome.

## 2 CASE PRESENTATION

A 32‐year‐old man was admitted to the emergency department with the chief complaint of loss of consciousness. He had drunk homemade alcoholic beverages few hours before and had no history of diabetes mellitus or hypertension. The patient underwent hemodialysis and received supportive care because of his life‐threatening condition. After 4 days, an ophthalmologist was asked to examine the patient because of the complaint of blurred vision. The examination revealed that he had poor light perception (PLP) on both eyes, but anterior and posterior segments of both eyes were within normal limits. With the clinical impression of optic neuropathy, he was treated with corticosteroid pulse (1 g/day) and intravenous EPO injection (20,000 IU/day) for 3 days. A single intravitreal injection of EPO (2000 IU) was also performed in both eyes under complete sterile condition and using different syringes to prevent infectious endophthalmitis, after containing a written informed consent. After 3 days, the corticosteroid pulse was changed to oral prednisolone (75 mg per day) for 2 weeks and then was tapered, and he was discharged from hospital with the VA of 20/800 (1 m counting finger) bilaterally, 10 days after his admission.

On the 10th day after discharge, the patient was examined for the second time. The general examination showed a VA of 20/800 in his both eyes. The relative afferent pupillary defect (RAPD) was negative bilaterally. The slit‐lamp examination (SLE) showed that the anterior segments and intraocular pressure (IOP) were normal for both eyes. Subsequently, the patient was injected with intravitreal EPO (2000 IU) in both eyes under complete sterile condition and using different syringes, once again. Two weeks later, his VA improved to 20/200 bilaterally and remained stable for 4 months.

**FIGURE 1:**
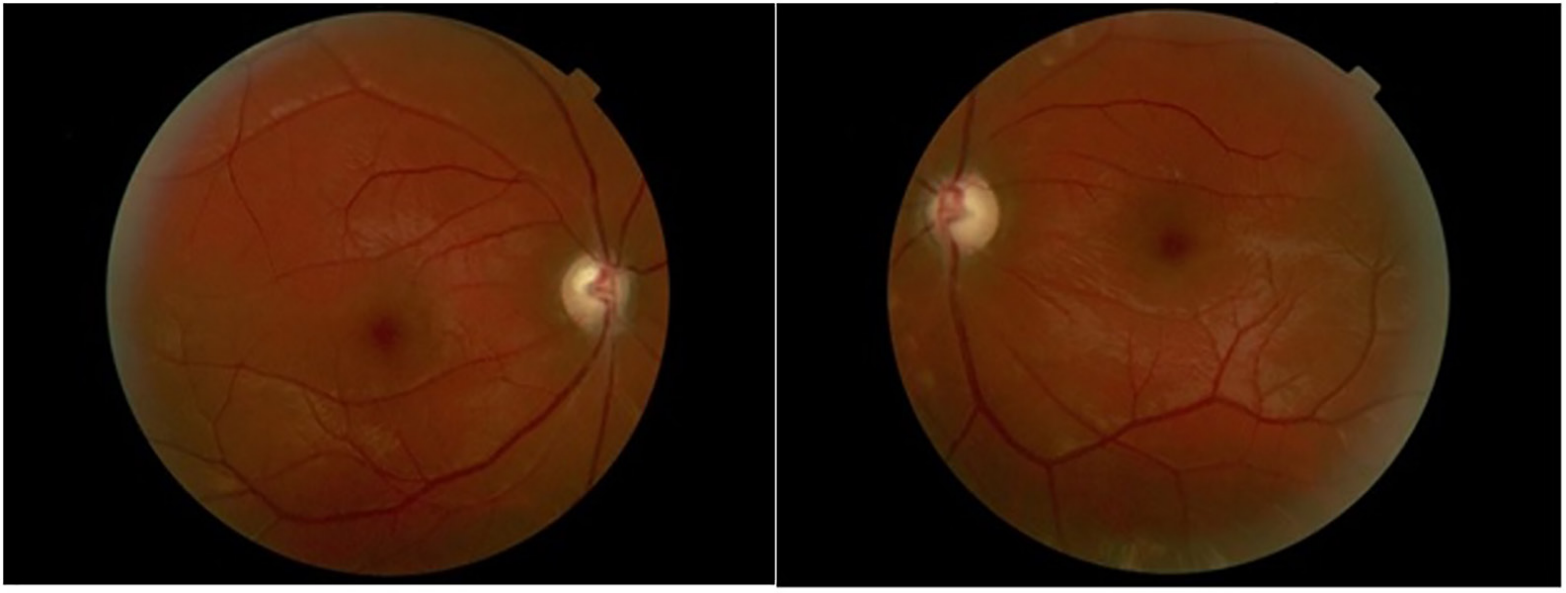
Color fundus photograph of both eyes. Fundus photograph revealed optic nerve atrophy and retinal nerve fiber layer loss in both eyes after 2 years follow‐up.

The follow‐up of the patient continued for 2 years after discharge. The last examination revealed that VA in both eyes had decreased to 20/320 (4 m counting finger) but RAPD was still negative bilaterally. The results of the SLE showed pale discs and retinal nerve fiber layer (RNFL) loss in both eyes (Figure 1).

## 3 DISCUSSION

Homemade alcohol was the source of methanol poisoning in our patient. This type of drink is the leading cause of methanol‐induced optic neuropathy in developing countries.^8^ Methanol's neuropathic effects include fundus problems, optic disc edema, and especially necrosis in the retrolaminar portion of the optic nerve that leads to retinal nerve fiber layer (RNFL) ischemia. RNFL ischemia is caused by oxidative stress that produces ROS and results in the necrosis of neurons, with necrosis eliciting the inflammatory response of the immune system.^9^

In our case, we administered corticosteroid pulses, due to their anti‐inflammatory effects and inhibition of demyelination process, go along with what Abrishami et al.^10^ suggested. However, Shams et al.^11^ indicated that methylprednisolone was not so effective in treatment of MION.

Erythropoietin seems to have both neuroprotective and neurotrophic effects. The possible mechanisms of EPO actions are reduction of ROS amount, following apoptosis and inflammation decreases. Besides EPO stimulates progenitor cell proliferation which can result in retinal nerve repairment in long‐time.^7^ Because of EPO's effects on cell stabilization and proliferation of progenitor cells, we used intravenous EPO injections simultaneously. In confirmation, Zamani et al.^12^ have shown that subcutaneous EPO may have strong but transient protective effects on MION at the early stages of the intervention which can represents neuroprotective effect of EPO. Another study confirms our conclusion that intravenous EPO can be used as an addition to the current treatments for patients with MION.^13^

Besides subcutaneous and intravenous administration of EPO, intravitreal injection may be more effective and have less systemic side effects such as hypertension, cardiovascular complications, and polycythemia.^14^ Modarres et al.^15^ declared that intravitreal EPO might be a safe and effective treatment for non‐arteritic anterior ischemic optic neuropathy. In addition, results of a case series in Egypt showed that intravitreal EPO injection could be an effective therapy for indirect traumatic optic neuropathy.^16^ Given the facts above, we decided to add intravitreal EPO injections to intravenous EPO and methylprednisolone for the first time.

Pakdel et al. and Pakravan et al. reported relatively higher improvements after their interventions in similar cases but our result was in line of which Zamani et al. reported.^12^, ^13^, ^17^ However, we noticed that VA improvement maintained in long‐term follow‐up. In our opinion, delay in beginning of specific intervention may be the underlying cause of our findings. If we had realized on the admission of the patient that his vision was affected, we could have started the treatment earlier, possibly with better long‐term results.

It seemed necessary that, the effect of intravitreal EPO injection on MION to be investigated with and without intravenous EPO and corticosteroid injections, in future controlled trials with larger sample sizes and longer term follow‐ups, to determine the effect more accurately.

## 4 CONCLUSION

In conclusion, we suggest that a combination usage of intravitreal and intravenous EPO in addition to intravenous pulses of corticosteroid could improve the visual outcomes in MION. However, in order to distribute it to whole cases, clinical researches must be carried out.

## AUTHOR CONTRIBUTIONS

**Mehran Rashidi Alavijeh:** Data curation; writing – original draft; writing – review and editing. **Sadegh Mazaheri‐Tehrani:** Conceptualization; data curation; writing – original draft; writing – review and editing. **Amir Sepehr Saffari:** Writing – original draft; writing – review and editing. **Mohammadreza Fazel:** Conceptualization; investigation; methodology; writing – review and editing. **Farhad Fazel:** Conceptualization; methodology; project administration; writing – review and editing.

## FUNDING INFORMATION

The authors would like to express that we have no funding received from any organization.

## CONFLICT OF INTEREST STATEMENT

The authors would like to express no conflict of interest.

## ETHICAL APPROVAL

The present study was approved by the Medical Ethics Committee of Isfahan University of Medical Sciences (IR.MUI.MED.REC.1399.726). The purpose of this report was completely explained to the participant and a written consent form was also obtained from the participants.

## CONSENT

Written informed consent was obtained from the patient for publication of this case report and any accompanying images. A copy of the written consent is available for review by the Editor‐in‐Chief of this journal.

## Data Availability

Data sharing not applicable to this article as no datasets were generated or analysed during the current study.
